# Exploring the Perceptions of Construction Workers and Senior Management Towards Mental Wellness Interventions Using Q-Methodology

**DOI:** 10.3390/ijerph22010052

**Published:** 2024-12-31

**Authors:** Evan Stoddard, Siddharth Bhandari, Fred Sherratt, Lauren Bone, Sloan Russell

**Affiliations:** Department of Civil, Environmental, and Architectural Engineering, University of Colorado, 1111 Engineering Drive, UCB 428, Boulder, CO 80309-0428, USA; siddharth.bhandari@colorado.edu (S.B.); fred.sherratt@colorado.edu (F.S.); lauren.bone@colorado.edu (L.B.); sloan.russell@colorado.edu (S.R.)

**Keywords:** mental health, construction industry, mental wellness interventions

## Abstract

The construction industry faces a significant crisis with rising suicide rates and mental health issues among workers. Addressing these challenges requires both systemic changes in work practices and improved access to mental wellness resources. However, there is limited research on the perceptions of both workers and senior management towards proposed solutions to reducing psychological distress experienced in occupational settings. Understanding these perceptions is crucial to determining the needs and acceptability of different wellness interventions. Thus, this study aimed to uncover preferences for validated wellness interventions in occupational settings by exploring the attitudes of 12 senior managers and 15 frontline workers using Q-Methodology. Findings revealed that frontline workers favored personalized wellness interventions tailored to their unique situations. Additionally, notable differences were identified between frontline workers and senior managers in their views on policies and practices aimed at reducing workload pressures and enhancing accountability and communication. The data also showed that current contracting and work practices potentially hinder the implementation of interventions perceived to be most beneficial by workers and senior managers. These results provide valuable insights for future research and offer guidance to organizations on designing, implementing, and communicating effective wellness interventions.

## 1. Background

Globally, construction workers are experiencing heightened levels of workplace stress [1], leading to increased absenteeism [2], growing opioid dependence [3], and a surge in suicide rates [4]. These alarming trends reflect the worldwide decline in mental health and wellness [5], but the construction industry faces additional challenges, as skilled labor shortages and an aging workforce exacerbate its struggle to maintain a healthy workforce.

Research into mental health within the construction, engineering, and management (CEM) sector is a growing field, primarily focused on identifying the underlying causes of poor mental health. Recent studies have systematically explored a wide range of stressors—both work-related and external—that adversely impact construction workers [6,7]. These include financial strain, excessive job demands, work-life imbalance, workplace violence, limited opportunities for career advancement, remote work locations, and lack of support [8]. Such stressors have been shown to detrimentally affect physical and mental health [9,10,11], increase susceptibility to addictions [12,13], and even contribute to suicidal ideation [14,15]. However, addressing these trends solely by mitigating symptoms of poor mental health overlooks the potential to proactively enhance mental well-being.

This study focuses on improving mental well-being, which, according to the dual-continua model of mental health [16], is conceptually distinct from the absence of mental illness. The dual-continua model posits that mental health and mental illness exist on separate, interrelated axes. While poor mental health refers to conditions such as anxiety, depression, or stress, mental well-being encompasses positive states of functioning, including resilience, purpose, and life satisfaction. Critically, an individual can experience high mental well-being despite living with mental illness, emphasizing the need for interventions that promote strengths rather than solely reduce symptoms. By focusing on mental well-being, this research seeks to foster resilience and enhance positive psychological states among workers.

As research uncovers the root causes of these stressors, the construction industry needs to examine systemic changes in how work is contracted and delivered [17]. Addressing these systemic challenges demands “whole-of-industry” solutions, involving organizational transformation and work redesign, supported by the collective efforts of contractors, clients, regulators, and trade partners [17,18,19,20]. While such systemic changes are vital, achieving the necessary consensus and coordinated action for these industry-wide transformations may require significant time. In the interim, this paper explores practical, evidence-based interventions that promote mental well-being while addressing the unique challenges faced by construction workers.

In the meantime, organizations are increasingly adopting individual-level preventive and therapeutic interventions to improve workers’ psychological and physical well-being. Unfortunately, there is limited academic research on the effectiveness and practicality of these interventions within the CEM sectors which poses challenges for the industry, especially resource-constrained companies. These organizations must carefully prioritize interventions they believe will deliver the most substantial positive impact with limited evidence. Additionally, differences in perspective between senior leadership and frontline workers—shaped by their differing lived experiences—can further complicate this prioritization. Consequently, this can lead to ad-hoc decision-making that may result in costly investments that either fail to meet frontline workers’ needs or lack effective implementation strategies.

This article aims to address this gap in the literature by (1) identifying and reviewing evidence-based individual-level wellness interventions, (2) comparing the perceptions of frontline workers and senior management regarding the perceived benefits of these interventions, and (3) analyzing senior management’s views on the practical feasibility of implementing these interventions. The study employs Q-Methodology to explore patterns of similarity and divergence in the ratings of wellness interventions by frontline workers and senior management. These findings will offer data-driven insights into which interventions should be prioritized while identifying areas for future research.

### Individual-Level Interventions to Improve Wellness in the Workplace

Mental well-being and mental wellness refer to the same overarching concept in this paper and are used interchangeably to describe positive psychological states such as resilience, purpose, and life satisfaction [21]. While the terminology remains a subject of debate in the literature, this study adopts a unified perspective to focus on promoting these positive outcomes through targeted workplace mental health interventions. In the workplace, this reflects an individual’s overall perception and experience of their working life [22]. While industry seeks to implement systemic changes at both organizational and sector levels, there has also been a simultaneous surge in efforts to equip individuals with the necessary tools for their own better mental health management. Research indicates that individual-level interventions are gaining significant traction in workplaces, encompassing programs such as employee assistance programs (EAPs), cognitive–behavioral interventions, peer-support systems, resilience training, relaxation and mindfulness programs, stress management training, lifestyle promotion, and health education [18,19,23,24]. These interventions are positioned as both preventative and supportive, aimed at bolstering psychological resilience by equipping individuals with the skills to recognize subclinical signs of mental illness among themselves or their colleagues.

The theoretical basis for individual-level interventions is grounded in the job demands-resources (JD-R) model, which suggests that workplace well-being is shaped by the balance between job demands and resources. Job demands involve the physical, cognitive, and emotional efforts required from individuals, while job resources help mitigate the strain these demands impose, enhancing motivation and job satisfaction [25]. Accordingly, individual-level interventions aim to improve workers’ psychological and physiological resilience through education, skill development, and access to resources, enabling them to better manage workplace demands. For example, mindfulness, relaxation, and resilience training equip individuals with strategies to prevent, manage, and recover from stress [26]. Similarly, peer-support systems foster a culture of openness and shared experiences, reducing stigma and reinforcing social support structures that enhance well-being [27]. Interventions that provide tangible skills have proven especially effective in promoting mental health [28]. A range of systematic reviews indicates that the evidence supporting individual-level interventions is generally positive, from relaxation techniques to stress management skill development [28].

It is also important to acknowledge that many academics and practitioners have criticized this approach to workplace wellness, arguing that it shifts the responsibility for maintaining mental well-being from the organization to the individual. Philosophically, it is contended that such interventions divert attention from the root causes of workplace stress by focusing on changing the individual rather than addressing systemic issues within the work environment [29]. Furthermore, much of the evidence on the effectiveness of individual-level interventions lacks the necessary external validity to be considered widely applicable across different work settings and contexts [24,30]. Finally, there is limited research demonstrating whether the benefits of these interventions are sustained over the long term among individuals with differing backgrounds [31].

Despite these drawbacks, prevailing evidence suggests there is a strong possibility that these interventions can make a positive difference, although the strength and duration of positive impact remains nebulous [31]. This consensus in work psychology literature is used as a foundation to ask and answer the following research questions:

1. Do frontline workers and senior managers share similar perceptions of which individual-level wellness interventions are beneficial?
2. Is there alignment in management’s perception of interventions perceived to be ‘beneficial’ and ‘practical’?

## 2. Research Method

To investigate how field and management personnel perceive the benefits and practicality of specific practices, Q-Methodology was employed. Q-Methodology is a technique suited for exploring subjective viewpoints which facilitates the systematic examination of individual perspectives on a given topic [32]. It enables the structured exploration of personal opinions by engaging participants in a process of prioritizing and categorizing their views, thus capturing diverse perspectives and identifying areas of consensus or divergence [33].

Q-Methodology begins with creating a set of statements or images, referred to as the Q-Sample, which participants (the P-Set) are asked to rank according to specific criteria set by the researchers [34]. This sorting exercise, known as the Q-Sort, results in a pattern that reflects the subjective views of the participants. The collected data are then analyzed using factor analysis to identify prevailing perceptions within the demographic group. Comparisons can be drawn between the sorting patterns of different groups, offering insights into shared or contrasting perspectives across various demographic cohorts [34].

### 2.1. Q-Set Generation

A literature review was performed using the framework proposed by [35] to identify individual-level wellness interventions that have been previously validated for use inside workplaces. The following steps were taken to complete a review of the literature.

#### Literature Search Strategy

Google Scholar was exclusively used for a comprehensive search across electronic databases. The key terms used in the literature search in conjunction with “workplace” and “mental health” were: “mental health workplace safety”, “security”, “social support”, “belonging”, “autonomy”, “flexibility”, “meaning”, “dignity”, “accomplishment”, “individual level”, “intervention”, “mindfulness”, “stress management”, “job demands”, and “learning”.

The studies reviewed in this paper focused on validating individual interventions across occupational domains. The selection criteria included scientific and experimental articles published in peer-reviewed journals, written in English, and with full-text access available. Articles published in languages other than English were excluded. Additionally, studies focusing on gaming-related interventions were omitted, as no quantitative evidence was found to support their application in the workplace. Research involving drug interventions or conducted in psychiatric settings was also excluded to maintain an appropriate occupational context. The review targeted interventions aimed at developing cognitive–behavioral skills among employees in the workplace, excluding those requiring input from medical practitioners. The search criteria for this review were initially established through a broad exploration of the mental health field. As the research focus was refined, a targeted collection of relevant interventions was conducted. The primary investigators applied the identified keywords systematically to the full text of articles, recognizing that the term “intervention” might not always be the central theme of the studies.

To enhance the content validity of the literature review findings, a panel of 12 construction industry professionals reviewed the proposed list of interventions and provided feedback. The panel members were qualified as industry experts, having at least five years of experience in the construction sector, with roles that involve providing direct mental health support to construction workers, such as safety professionals and mental health specialists for their respective companies. To capture the diversity of working environments and workforce dynamics, the experts represented various sectors with heavy civil, vertical construction, energy production, utilities, and trade union sectors of the construction industry. Table 1 summarizes individual-level interventions tested within occupational contexts. Individual-level interventions are targeted strategies that organizations implement to address the mental well-being of employees on a personal or small-group basis. These interventions are distinct from organizational-level approaches, which affect all workers in a company (e.g., universal pay raises), and industry-level interventions, which involve regulatory or systemic shifts (e.g., sector-wide overtime limits). The individual-level interventions highlighted in Table 1 are selected based on their applicability in workplace contexts, with a focus on improving workers’ psychological resources and resilience. Their adaptability makes them especially relevant in the construction industry, where roles and environments vary significantly.

In order to distil the interventions into a data set suitable for the Q-Sort, the research team refined the full list of interventions by combining similar approaches and incorporating additional procedures commonly employed by construction companies. Distinctions were also made for certain interventions, such as lifestyle changes and support groups, specifying whether they were optional or mandatory for workers. This differentiation aimed to assess the perceived benefits of mandatory participation, particularly for workers who might otherwise avoid mental health interventions due to social stigma. The inclusion of mandatory options sought to capture perceptions on the impact of universal participation in mental wellness programs. Overall, this process resulted in a final list of 21 interventions as shown in Table 2.

### 2.2. Q-Sorting Process

Data was collected from six professional groups: three groups of frontline workers and three groups of senior management. The data was collected across United States and Canada in early 2024. To minimize the potential for a bandwagon effect, each group had a maximum of five participants [121]. The senior management groups consisted of 12 individuals, divided equally across three groups. The twelve individuals represented different construction organizations. The three frontline worker groups had one group of roofers, a group of laborers, and one of line workers. Each group consisted of participants from the same organization within their group. However, the three groups themselves represented different organizations. Although the sample size is relatively small, which presents some limitations, Q-Methodology studies typically do not require a large number of participants [34,122]. Instead, participants are purposively selected to capture a wide range of opinions and experiences, enabling in-depth exploration and laying the groundwork for future research based on the diversity of perspectives. Therefore, the authors focused on including senior managers involved in designing and implementing mental health interventions in their organizations. Additionally, the frontline workers in this study represent various sectors within the construction industry. These selected frontline workers completed the Q-Sort process in a private room away from any of their supervisors to keep their responses confidential and to prevent any potential reprimand from their employers for the answers given during the testing. The sessions were also completed at work and were agreed to by their employers to ensure that the field workers who took part in the study did not face any reprimands for lost work time or time away from the job site.

The Q-Sorting process required participants to place the individual interventions on a quasi-normal distribution curve, resulting in an objective ranking of the subjective data. The 21 interventions (from Table 2) were printed on index cards to enable the participants to easily lay out the interventions for discussion and final placement in the distribution. The participant groups were instructed to rank the interventions on a scale ranging from −2 to +2, with verbal descriptors of “Least Beneficial”, “Minimally Beneficial”, “Somewhat Beneficial”, “Highly Beneficial”, and “Extremely Beneficial”. This wording was intentionally selected to recognize that, while all interventions could offer some level of benefit, the degree of benefit may vary. Mathematically, the forced distribution curve necessitated discussions amongst the group to carefully evaluate each intervention before placing it in an agreed place on the curve. This structured approach to ranking has been effectively utilized in previous research to explore mental models in mental health and construction, engineering, and management (CEM) studies, providing insights into participants’ subjective judgments [123].

Each group was allotted up to 90 min to complete the sorting process, during which researchers observed participants’ decisions and provided clarification on the scope of each intervention as needed to maintain consistency across the group. Facilitators also took notes on participant discussions to capture contextual insights, although these discussions were not audio-recorded. This decision was made to foster open and honest reflections about the applicability of the interventions in the workplace. The only difference between the two professional groups (field workers and senior management) was that field workers did not evaluate the perceived practicality of the interventions.

Senior managers used a similar rating system (−2 to +2) to evaluate the practicality of each intervention, with the categories labeled “Not Possible”, “Very Difficult”, “Difficult”, “Easy”, and “Very Easy”. After completing the Q-Sort exercise, the scores for each of the 21 interventions were recorded for each individual group and analyzed using univariate statistical methods.

## 3. Findings

A total of six Q-Sorts were conducted, one for each group of participants. To obtain the final results, the Q-Sorts from the three frontline worker groups were aggregated to produce a composite Q-Sort for frontline workers. Similarly, the Q-Sorts from the three senior management groups were aggregated to create a composite Q-Sort for senior managers. This process resulted in two final Q-Sorts, representing the overall rankings of each intervention for frontline workers and senior managers.

Table 3 presents the final rankings of interventions as prioritized by senior managers, based on the composite Q-Sort results. These rankings reflect the aggregated preferences of the senior management groups, with higher ranks indicating greater perceived importance or practicality. Similarly, Table 4 displays the final rankings of interventions prioritized by frontline workers, based on their composite Q-Sort results. These rankings capture the collective preferences of the frontline worker groups, providing a comparative perspective to the priorities identified by senior managers.

The rankings in both tables were determined through the Q-Sorting process and represent the relative prioritization of interventions. It is important to note that these rankings do not correspond directly to any specific column in the tables but are instead derived from the overall sorting and weighting exercise conducted during the Q-Sort.

In addition to evaluating the perceived benefit, the three groups of senior managers were asked to complete an additional Q-Sort to assess the perceived practicality of each intervention. The results of this Q-Sort are presented in Table 5.

The study analyzed Spearman correlations to assess agreement on the rankings of mental health interventions among three senior manager groups and three worker groups (Roofers, General Laborers, and Linemen & Field Safety) within the construction industry. Results indicated low to moderate intra-group consistency for senior managers, with correlations ranging from 0.18 to 0.32, none of which were statistically significant (*p* > 0.05). Among worker groups, correlations ranged from 0.26 to 0.45, with a significant alignment observed between Roofers and General Laborers (r = 0.45, *p* = 0.04). These results have been summarized in Table 6 and Table 7.

Finally, an inter-group comparison (i.e., comparing the aggregated rankings) across senior managers and workers showed no significant correlations (r = 0.66, *p* > 0.05), suggesting the alignment in perceptions was not strong enough to be considered statistically significant across the groups.

Overall, these findings indicate that while some alignment exists among workers, particularly between Roofers and General Laborers, senior managers exhibited more diverse perspectives. The results point to a potential gap in the prioritization or understanding of mental health needs between management and frontline workers, emphasizing the need for tailored strategies to address group-specific views in implementing workplace mental health programs.

## 4. Discussion

### 4.1. Perceived Benefit

The analysis revealed clear differences between frontline workers and senior managers in their perceptions of the benefits of mental health interventions. Among frontline workers, *Trainings Focused on Lifestyle Changes to Promote Wellness* and *Expanded Health Coverage* were the top-rated interventions, reflecting a shared belief in the value of providing tangible support to address mental health. Additional interventions such as *Optional Lifestyle Changes*, *Accountability and Communication*, *Leadership Engagement*, and *Changes to Financial Compensation* were also rated favorably, suggesting a preference for programs that not only demonstrate genuine organizational commitment but also empower workers to access resources according to their individual needs.

In contrast, senior managers focused on interventions addressing stressors like Job Demand and Financial Insecurity, with *Workload Reduction* emerging as the top-rated intervention. However, this approach did not resonate with frontline workers, who viewed workload reduction less favorably, potentially due to concerns over lost income from reduced overtime and the added pressures to maintain productivity targets. Furthermore, senior managers rated *Leadership Engagement* and *Accountability and Communication* lower than frontline workers did, indicating a possible underestimation of the importance that workers place on active leadership in mental health initiatives.

Despite some alignment between the two groups—both ranked *Downtime at Work*, *Mandatory Lifestyle Changes*, and *Mandatory Support Groups* as the least beneficial—these results underscore fundamental differences in how management and workers believe work-related stressors should be managed. The shared preference for maintaining autonomy over mental wellness decisions suggests a need for interventions that respect individual choice.

The Spearman correlation analysis highlighted the variability not only between groups (senior managers vs. frontline workers) but also within groups. Inter-group comparison revealed no significant correlation between the rankings of senior managers and frontline workers (r = 0.66, *p* > 0.05), indicating distinct perceptions of mental health interventions’ value between these organizational roles. Among senior managers, low to moderate correlations (ranging from 0.18 to 0.32) indicated inconsistent viewpoints across the three groups, none of which were statistically significant. Worker groups showed slightly higher correlations (0.26 to 0.45), with a significant alignment only between Roofers and Laborers (r = 0.45, *p* = 0.04). Given the inherently personal nature of mental health, it is not surprising that these findings reflect varying perceptions even within seemingly homogeneous demographic groups. The diverse lived experiences of individuals suggest that shared group characteristics do not necessarily translate into uniform views on mental health interventions. This variability indicates that individual-level interventions may have limited external validity, as the effectiveness and relevance of such programs can vary widely across different contexts and personal circumstances. Mental health strategies should account for these differences and prioritize flexibility and adaptability in their design to better accommodate the unique needs of each individual. Future investigations with more robust data pools should seek to cross-validate these findings.

### 4.2. Perceived Practicality

For senior managers, the most practical interventions were those involving structured planning and informational resources, such as *Creating a Formal Written and Strategic Plan*, *Personalized Resources for Workers*, and *Online-Based Peer Support Groups.* The analysis also revealed a disconnect between perceived benefit and practicality, particularly for interventions like *Workload Reduction* and *Expanded Health Coverage*, which were rated highly for benefit, but most participants felt there were significant barriers to their practical implementation. This gap underscores the challenges associated with adopting high-impact interventions that may be constrained by financial limitations or operational complexities. The lack of any correlation between the ratings for perceived benefit and perceived practicality among senior managers (r = 0.03, *p* > 0.05) further underscores this point.

To address both practical considerations and employee needs, construction companies must ensure that mental health initiatives are not only feasible but also effective in meeting the unique demands of the industry. Bridging the gap between perceived benefit and feasibility will require tailoring interventions to the diverse needs of the workforce while accounting for practical limitations. This approach involves balancing meaningful mental health support with the realities of financial and operational constraints.

## 5. Implications

The findings suggest that construction companies should prioritize mental health interventions that emphasize choice and flexibility, such as *Optional Lifestyle Changes* and *Expanded Health Coverage*, which received more favorable ratings than mandatory programs. Additionally, the practicality results indicated that senior managers favored interventions that involve structured planning and informational resources, such as *Creating a Formal Written and Strategic Plan* and *Personalized Resources for Workers*. These preferences reflect a tendency to prioritize solutions that are administratively feasible and less resource-intensive, but which may not yield desired levels of positive impact.

Furthermore, overly simplistic solutions (e.g., workload reduction, downtime at work) often proffered within the CEM literature may not be perceived positively by relevant stakeholders. It is essential to discuss the unintended consequences associated with each intervention; any proposed intervention must be reported in the literature with detailed limitations to reflect the due diligence warranted by health-related interventions. Additionally, the findings underscore the practicality of individual-level interventions in construction contexts, where the variability in job roles and environments makes tailored approaches more feasible than systemic interventions. However, their effectiveness may be limited without complementary organizational-level changes that address systemic factors, such as workload demands or inadequate managerial support. This observation aligns with broader critiques in the literature, cautioning against reliance on individual-level strategies alone to tackle complex workplace challenges.

Finally, the absence of a correlation between perceived benefit and practicality (r = 0.03, *p* > 0.05) underscores the need for a careful evaluation of both the anticipated impact and the feasibility of interventions. Programs that appear valuable on paper may not translate into actionable solutions without considering practical barriers. Bridging the gap between benefit and feasibility will involve designing interventions that can be realistically implemented within the constraints of budget, time, and workforce resources. Tailoring programs to accommodate practical limitations while still meeting employee needs will be essential for successful mental health initiatives.

## 6. Limitations

While adopting Q-Methodology provided valuable insights into the perceived benefit and practicality of mental health interventions among frontline workers and senior managers across various construction sectors, certain limitations must be acknowledged. First, this study did not evaluate the actual outcomes or effectiveness of the 21 interventions ranked; instead, it offers a framework to guide future research in prioritizing interventions deemed to have the highest potential benefit by key stakeholders. Casual examinations need to be conducted to ascertain the effectiveness of each proposed intervention. The findings presented here serve as a preliminary step in identifying which interventions warrant further empirical investigation rather than providing conclusive evidence of their impact. Secondly, as with opinion-based studies, group-related activities can introduce biases such as dominant voices influencing the rankings or groupthink [124]. While controls such as supervision from research facilitator and maintaining small group sizes were included to minimize biases associated with group dynamics, the elimination of such biases was not possible, thereby potentially compromising the ratings. Thirdly, the sample size, though not required to be large in studies leveraging Q-Methodology, was relatively small, which could be remedied in future explorations. Fourth, to maintain anonymity of participants involved in the study, the authors did not collect demographic data to perform multivariate analysis, an omission which should be addressed in future studies. Fifth, it is also important to note that some of the industry experts consulted during the literature review overlapped with the respondent groups participating in the Q-Sort process. While the experts’ feedback was limited to validating the comprehensiveness of the list of interventions and did not involve judging their quality, this overlap may have introduced unintended bias in the data. Future studies should cross-examine findings by ensuring independent validation of intervention lists and respondent feedback to minimize potential influence and enhance the robustness of the conclusions. Sixth, and finally, this study primarily utilized Google Scholar for the literature review, which has been shown to outperform other databases in terms of comprehensive coverage [125]. However, this was not a systematic literature review, and some niche publications indexed in databases such as Web of Science and Scopus may have been excluded. Future research should incorporate multiple databases to ensure broader coverage and enhance rigor.

These limitations suggest that while the current study offers a robust framework for understanding stakeholder perceptions, further research is needed to validate the findings through more individualized approaches and empirical testing of the ranked interventions.

## 7. Conclusions

This study identified mental health interventions that construction industry stakeholders perceive as most beneficial, highlighting the importance of providing workers with options to address their mental health in a personalized manner. The top-rated interventions included increasing access to a variety of mental health resources, enhancing financial compensation, and raising awareness around mental health issues. These findings suggest a preference for strategies that empower workers to take control of their mental well-being, rather than prescriptive or mandatory approaches.

However, there were notable differences in opinion between frontline workers and senior managers, particularly regarding *Workload Reduction and Accountability and Communication* as potential mental health interventions. While senior managers viewed workload reduction as a way to alleviate stress, workers clearly did not see it the same way—underscoring the need for nuanced approaches that balance workload management with economic realities. The divergence in perceptions of accountability also suggests that workers may experience unaddressed workplace issues differently to how senior managers perceive them. A significant gap emerged between interventions perceived as having the highest potential benefit and those deemed most practical to implement. This disconnect indicates that many senior managers may perceive substantial barriers to enacting meaningful change swiftly, particularly when faced with financial or logistical challenges.

Overall, this exploratory study provides a foundation for future research by prioritizing interventions that are considered beneficial by key stakeholders in the construction industry. The framework established here can serve as a baseline for subsequent confirmatory studies, aimed at empirically testing the effectiveness of the interventions identified as having the greatest potential impact. Future studies could expand on this research by incorporating structured feedback mechanisms to capture participants’ reflections on their rankings and exploring psychological responses to interventions. Additionally, breaking down broad interventions, such as flexible work arrangements, into more specific categories could provide deeper insights into their perceived benefits and practicality while maintaining methodological rigor.

## Figures and Tables

**Table 1:** Summary of individual-level interventions from the literature.

Intervention	Definition	Potential Benefits	Citations
Peer Support	Brings together people with similar stressors for mutual support.	Reduces symptoms of depression, anxiety, and other mental health conditions. Improves physical and emotional well-being. Provides opportunities for skill-building and community integration.	[36,37,38]
Informational Websites	Employees use a reputable website to understand mental health issues on their own.	Ease of access to mental health information. Improves mental well-being and teaches individuals how to handle mental health emergencies.	[39,40,41]
Online Screening Tools	Employers offer online tests that individuals can use to evaluate their personal mental health.	Access to healthcare providers which allows individuals to monitor their mental health over time and encourages seeking help.	[39,42,43,44]
EAP/EFAP Healthcare Access	Providing employees with healthcare, including mental health resources (e.g., therapists).	Reduces symptoms of depression, anxiety, and stress. Decreases absenteeism and helps manage work–life stress. EAPs are cost-effective options.	[39,45,46,47]
Self-Directed Therapy	Guiding employees to websites designed to help improve their mental health independently.	Reduces symptoms of depression and anxiety. Empowers individuals to manage their mental health at their own pace and is more affordable than traditional therapy.	[39,48,49,50,51]
Flexible Work Arrangements	Allows workers to attend work remotely when possible and plan time off in advance.	Reduces stress from commuting, decreases burnout, improves work–life balance, and increases productivity.	[17,52,53,54,55]
Synchronous Leisure	Provides flexibility over work hours.	Reduces stress, improves mood, enhances job satisfaction, and builds trust among coworkers. Encourages healthy activities and reduces cognitive decline.	[17,56,57,58,59]
Work-Hour Reductions	Reduces work hours per week, day, or year.	Improves sleep, reduces stress, and promotes a better work–life balance.	[17,60,61,62]
Recovery	Encourages employees to take breaks to improve well-being.	Decreases stress and fatigue, improves physical health and sleep, reduces burnout and depression, and increases productivity and engagement.	[17,63,64,65,66]
Right to Disconnect	Provides a specific time during which employees are not required to respond to work communications.	Reduces burnout and improves job satisfaction.	[17,67,68]
Physical Activity/Yoga	Offers group yoga or aerobic classes weekly.	Reduces symptoms of depression, enhances emotional well-being, improves stress management, and boosts mental resilience.	[69,70,71,72,73,74]
Progressive Muscle Relaxation	Involves tensing and relaxing muscles as a relaxation technique.	Improves focus, sleep quality, body awareness, and productivity. Acts as complementary therapy for conditions like hypertension and fibromyalgia.	[75,76,77,78]
Resiliency Apps	Use of apps that help individuals manage stress and overcome challenges.	Reduces stress and anxiety. Convenient and accessible with personalized, continuous support.	[79,80,81,82,83,84,85,86]
Mental Health First Aid	Training to identify and respond to mental health crises.	Increases mental health literacy, crisis management skills, and reduces stigma.	[87,88,89]
Online Support Groups	Online groups for people with similar stressors to support each other.	Reduces symptoms of depression and anxiety. Improves quality of life, offers social support, and is cost-effective and accessible.	[48,90,91,92]
Health Screening	Screening for stress symptoms like hypertension and ambulatory conditions.	Prevents suicide, reduces symptom severity, promotes resilience, and provides early intervention.	[81,93,94,95,96,97]
Anger Management Training	Focuses on helping individuals regulate their emotions and control anger.	Reduces stress, improves self-esteem and relationships, and enhances work performance and communication skills.	[98,99,100,101,102,103,104,105,106]
Cognitive Behavior Therapy	Breaks down overwhelming issues into manageable parts.	Reduces anxiety symptoms, builds coping skills, and offers customizable, long-term effectiveness.	[107,108,109,110,111]
Relaxation Response Training	Provides practices to induce a deep state of relaxation.	Lowers blood pressure, improves sleep quality, enhances cognitive function, and improves decision-making and stress management.	[112,113,114,115,116,117,118]
Resiliency Training	Program to improve ability to cope and recover from adversity.	Reduces symptoms of depression and anxiety, improves coping skills, and increases self-efficacy and emotional regulation.	[26,81,119,120]

**Table 2:** Final Interventions List.

Intervention	Description
Personalized Resources	Newsletters, informational websites, informational packets.
Workplace Counseling Services	In-house mental health professionals.
Trainings on Awareness of Mental Health Issues	Learning when and how to seek help with mental health.
Online-Based Peer Support Group	Online meetings of peers to discuss mental health topics.
Workload Reduction	Policy and procedure changes to maximise amount of time able to work.
Mandatory Lifestyle Changes	Mandatory social gatherings, healthy food, fitness facility usage, etc.
Flexible Work Arrangements	Autonomy at work, flexible work hours, and online options.
Downtime at Work	Time during day to rest and not be required to work.
Technological Interventions	Interactive online educational tools, online therapy, and AI-based resources.
Optional Health Assessments	Physical and mental health screenings at the workplace and paid for by the company.
Mandatory Support Groups	Mandatory support group attendance by workers to discuss mental health challenges.
Training on Lifestyle Changes	Education on changes to lifestyle to promote physical and mental wellness.
Optional Lifestyle Changes	Optional social gatherings, healthy food, fitness facility usage, etc.
Expanded Health Care Coverage	Mental health related services covered by insurance for workers and their families.
Leadership Engagement	Upper management are engaged and involved in mental health focus for the company.
Training on Policies, Programs, and Procedures	Education for workers on the company’s policies and programs on mental health.
Accountability and Communication	Repercussions for policy or procedure violations by employees.
Changes to Financial Compensation	Revision to pay cycles, increased compensation, bonuses, and incentives.
Change Physical Attributes of Work	Better walkability of sites, and improved rest areas for workers.
Formal Written and Strategic Plan	Company plan for addressing mental health and wellness.
Incidental Support Groups	Support groups put in place after a potentially traumatic event takes place.

**Table 3:** Q-Sort perceived benefit (senior managers).

Interventions	Average Score	Median Score	Standard Deviation
Changes to Financial Compensation	1.0	1.0	0.8
Workload Reduction	1.0	2.0	1.4
Expanded Health Coverage	1.0	1.0	0.0
Training (Awareness of MH issues)	1.0	1.0	0.0
Training (Lifestyle changes to promote wellness)	1.0	1.0	0.8
Flexible Work Arrangement/Autonomy at Work	0.3	0.0	1.2
Optional Health Assessment (at work, paid by company)	0.3	0.0	0.5
Change Physical Attributes of Work (walkability, rest areas)	0.3	1.0	0.9
Formal Written and Strategic Plan	0.7	0.0	0.9
Leadership Engagement (focused on MH)	0.7	0.0	0.9
Optional Lifestyle Changes (social gatherings, healthy food, fitness facility)	0.7	0.0	0.9
Workplace Counseling Services	0.3	0.0	1.2
Accountability and Communication	−0.3	0.0	0.5
Technological Interventions (Interactive tools for education and therapy, AI-based)	−1.0	−1.0	0.8
Training (Policies, Programs and Procedures)	−1.0	−1.0	0.8
Incidental Support Groups	−0.7	−1.0	0.5
Downtime at Work	−1.3	−1.0	0.5
Mandatory Lifestyle Changes	−1.0	−2.0	1.4
Mandatory Support Groups	−2.0	−2.0	0.0
Online-Based Peer Support Group (W/coworkers)	−0.7	0.0	0.9

**Table 4:** Q-Sort Perceived Benefit (Frontline Workers).

Interventions	Average Score	Median Score	Standard Deviation
Expanded Health Coverage	1.3	1.0	0.5
Training (Lifestyle changes to promote wellness)	1.3	2.0	0.9
Changes to Financial Compensation	1.0	1.0	0.8
Leadership Engagement (focused on MH)	1.0	1.0	0.0
Optional Lifestyle Changes (social gatherings, healthy food, fitness facility)	1.0	1.0	0.8
Accountability and Communication	1.0	1.0	0.8
Training (Awareness of MH issues)	0.7	1.0	0.5
Optional Health Assessment (at work, paid by company)	0.7	0.0	0.9
Change Physical Attributes of Work (walkability, rest areas)	0.3	0.0	0.5
Workplace Counseling Services	0.3	0.0	0.5
Incidental Support Groups	0.3	1.0	0.9
Online-Based Peer Support Group (W/coworkers)	0.0	0.0	1.6
Formal Written and Strategic Plan	0.0	−1.0	1.4
Technological Interventions (Interactive tools for education and therapy, AI-based)	−0.3	0.0	0.5
Training (Policies, Programs and Procedures)	−0.3	−1.0	0.9
Flexible Work Arrangement/Autonomy at Work	−0.7	−1.0	0.5
Personalized Resources (newsletter, info websites, info packets)	−0.7	−1.0	0.5
Workload Reduction	−1.0	−1.0	0.8
Mandatory Lifestyle Changes	−1.7	−2.0	0.5
Downtime at Work	−1.3	−2.0	0.9

**Table 5:** Q-Sort perceived practicality.

Interventions	Average Score	Median Score	Standard Deviation
Formal Written and Strategic Plan	1.7	2.0	0.5
Personalized Resources (newsletter, info websites, info packets)	1.3	2.0	0.9
Online-Based Peer Support Group (W/coworkers)	1.3	1.0	0.5
Flexible Work Arrangement/Autonomy at Work	1.0	−1.0	0.8
Training (Awareness of MH issues)	1.0	1.0	0.8
Training (Lifestyle changes to promote wellness)	1.0	1.0	0.8
Incidental Support Groups	1.0	1.0	0.0
Training (Policies, Programs and Procedures)	1.0	1.0	0.8
Leadership Engagement (focused on MH)	0.3	0.0	0.5
Optional Lifestyle Changes (social gatherings, healthy food, fitness facility)	0.7	0.0	0.9
Optional Health Assessment (at work, paid by company)	0.0	0.0	0.8
Accountability and Communication	0.0	0.0	0.8
Change Physical Attributes of Work (walkability, rest areas)	−0.7	−1.0	1.2
Expanded Health Coverage	−0.7	−1.0	0.5
Mandatory Support Groups	−0.3	0.0	0.5
Technological Interventions (Interactive tools for education and therapy, AI-based)	−1.0	−1.0	0.8
Workplace Counseling Services	−1.0	−1.0	0.8
Downtime at Work	−1.0	−1.0	0.8
Workload Reduction	−1.3	−1.0	0.5
Mandatory Lifestyle Changes	−1.3	−1.0	0.5

**Table 6:** Inter-group correlations (senior managers).

	Group 1	Group 2	Group 3
Group 1	1		
Group 2	0.30	1	
Group 3	0.32	0.18	1

**Table 7:** Inter-group correlations (workers; * denotes statistical significance).

	Group 1	Group 2	Group 3
Group 1 (Roofers)	1		
Group 2 (Laborers)	0.45 *	1	
Group 3 (Lineworkers)	0.42	0.26	1

## Data Availability

Some or all data, models, or code generated or used during the study are proprietary or confidential in nature and may only be provided with restrictions.
